# In Pursuit of Novel Markers: Unraveling the Potential of miR-106, CEA and CA 19-9 in Gastric Adenocarcinoma Diagnosis and Staging

**DOI:** 10.3390/ijms25147898

**Published:** 2024-07-19

**Authors:** Adrian Boicean, Ioana Boeras, Sabrina Birsan, Cristian Ichim, Samuel Bogdan Todor, Danusia Maria Onisor, Olga Brusnic, Ciprian Bacila, Horatiu Dura, Corina Roman-Filip, Maria Livia Ognean, Ciprian Tanasescu, Adrian Hasegan, Dan Bratu, Corina Porr, Iulian Roman-Filip, Bogdan Neamtu, Sorin Radu Fleaca

**Affiliations:** 1 Faculty of Medicine, Lucian Blaga University of Sibiu, 550169 Sibiu, Romania; adrian.boicean@ulbsibiu.ro (A.B.); cristian.ichim@ulbsibiu.ro (C.I.); ciprian.bacila@ulbsibiu.ro (C.B.); horatiu.dura@ulbsibiu.ro (H.D.); corina.roman@ulbsibiu.ro (C.R.-F.); maria.ognean@ulbsibiu.ro (M.L.O.); ciprian.tanasescu@ulbsibiu.ro (C.T.); adrian.hasegan@ulbsibiu.ro (A.H.); dan.bratu@ulbsibiu.ro (D.B.); radu.fleaca@ulbsibiu.ro (S.R.F.); 2 Molecular Biology Laboratory of the Applied Ecology Research Center, Faculty of Sciences, Lucian Blaga University of Sibiu, 550012 Sibiu, Romania; 3 Faculty of Social Sciences, Lucian Blaga University of Sibiu, 550012 Sibiu, Romania; 4 Department of Gastroenterology, University of Medicine, Pharmacy, Science, and Technology of Targu Mures, 540142 Târgu Mures, Romania; halalisan5@yahoo.com (D.M.O.); brusnic_olga@yahoo.com (O.B.); 5 County Clinical Emergency Hospital of Sibiu, 550245 Sibiu, Romania; 6 Department of Neurology, “George Emil Palade” University of Medicine, Pharmacy, Sciences and Technology, 540136 Targu Mures, Romania; 7 Pediatric Research Department, Pediatric Clinical Hospital Sibiu, 550166 Sibiu, Romania; bogdan.neamtu@ulbsibiu.ro

**Keywords:** gastric cancer, biomarkers, miRNA, screening, carcinoembryonic antigen, carbohydrate antigen 19-9

## Abstract

Gastric cancer stands as the fourth leading cause of cancer-related deaths globally, primarily comprising adenocarcinomas, categorized by anatomic location and histologic type. Often diagnosed at advanced stages, gastric cancer prognosis remains poor. To address the critical need for accurate tumoral markers for gastric cancer diagnosis, we conducted a study to assess classical markers like CEA and CA-19-9 alongside the novel marker miR-106. Our investigation revealed distinct dynamics of these markers compared to non-cancerous groups, although no disparities were observed across different disease stages. Univariable and multivariable logistic regression analyses demonstrated that elevated levels of miR-106, CEA and CA 19-9 were predictive of a positive histopathological exam, with the respective odds ratios of 12.032 (95% CI: 1.948–74.305), 30 (95% CI: 3.141–286.576), and 55.866 (95% CI: 4.512–691.687). Subsequently, we utilized predicted probabilities from regression models to construct receiver operating characteristic (ROC) curves, identifying CA 19-9 as the optimal predictor for gastric adenocarcinoma diagnosis when considering age and gender, with an area under the curve (AUC) of 0.936 (*p* < 0.001). Hence, classical markers exhibit superior performance compared to the novel marker miR-106 in predicting gastric adenocarcinoma.

## 1. Introduction

Gastric tumorigenesis is a multistep process involving a series of epigenetic and genetic alterations. Knowledge about the dynamic molecular variations underlying gastric carcinogenesis has an important role for exploring the molecular mechanisms in gastric cancer, which is the sixth leading type of cancer worldwide and the fourth type of cancer in terms of mortality rate according to GLOBOCAN data 2020 [1,2,3,4].

The therapeutic management of gastric neoplasms represents a challenge, due to its limited curability possibilities and, therefore, any possibility of early detection ought to be exploited. The disease becomes symptomatic only in advanced stages, so multidisciplinary approaches are important to diagnose gastric cancer in early stages. The goal of the treatment in gastric cancer is to increase survival by curative surgery, but this requires effective screening using both tumor and endoscopic markers [5,6,7,8].

The importance of early detection of gastric cancer should remain a priority in current research, as it can yield multiple benefits for patients and also for the healthcare system. Thus, there is a necessity to search for molecular biomarkers that can be used for the detection of gastric cancer and approach the cellular and molecular modifications that lead to cancer development [9,10,11,12]. Early detection would involve endoscopic therapy and could prevent surgery, chemotherapy and improve patients’ survival rate and lower complications following gastric resection [12,13,14].

Due to the necessity of surpassing current research boundaries, recent studies are seeking novel tumor markers to aid in the earliest possible identification of gastric cancer. In this regard, there is a growing focus on miRNAs (particularly miR-106) in plasma/gastric juice or other fluids to establish non-invasive ways to detect early-stage gastric cancer, as potential biomarkers that can add specificity to the traditional markers CEA and CA 19-9, with many biological benefits and clinical potential. CEA and CA 19-9 are among the most frequently utilized tumor markers in gastric cancer diagnosis and monitoring. Moreover, previous studies show that plasma CA 19-9 and CEA levels are correlated with the TNM stage (*p* < 0.004) and survival rate (*p* < 0.001). While traditional biomarkers play a crucial role in monitoring disease post-resection, their sensitivity for screening gastric cancer is often deemed insufficient [10,15,16].

Micro-RNA molecules play a crucial role in gastric neoplasms by regulating gene expression. Due to their tissue specificity, they serve as reliable biomarkers for tumor progression [17,18,19].

While extensive research has delved into the molecular dysregulation of miR-106 in cancer, few studies have explored its association with cancer stages, tumor extension, lymph node involvement and distant metastasis. MiR-106 is linked to tumor progression and proliferation, often correlated with a negative prognosis in many cancers. Its upregulation has been observed in gastric cancer, as well as breast and colorectal cancers [20,21]. In the light of these modern findings, it is mandatory to emphasize the importance of changes in miR-106 in gastric cancer, as a method for personalized therapeutic management for patients and as a better screening tool [22,23,24,25,26,27,28,29,30].

The primary objective of this study was to evaluate and compare the effectiveness of miR-106 extracted from gastric juice with the levels of CEA and CA 19-9 in serum in predicting the presence of gastric adenocarcinoma through histopathological examination. Additionally, the study aimed to investigate the correlations between these three markers and various clinicopathological characteristics such as tumor size, lymph node involvement, and the presence of metastatic disease.

## 2. Results

**Figure 1:**
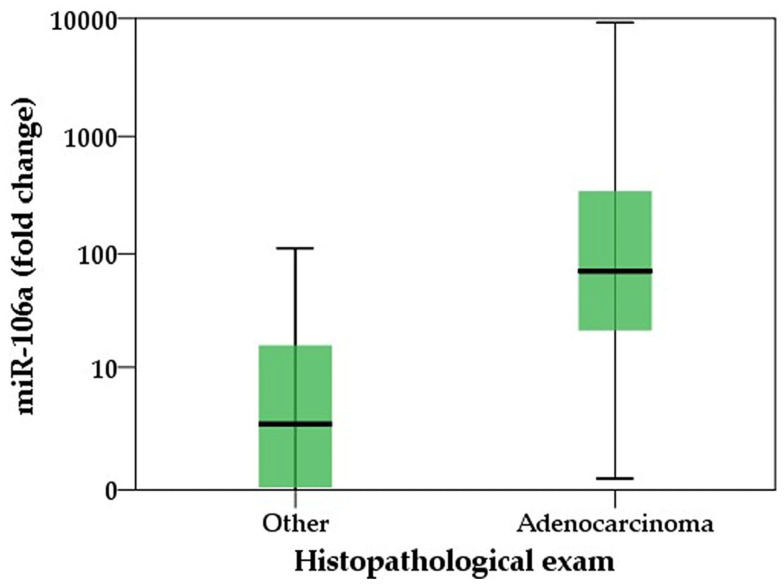
Distribution of miR-106a fold change between confirmed cases of gastric adenocarcinoma and controls; Mann–Whitney U test, *p* = 0.001.

For this study, we included a total of 38 patients divided into two groups: 22 patients diagnosed with gastric cancer, whereas the control group consists of 16 patients presenting with other gastric issues but did not have cancer. General characteristics of the patients are shown in Table 1. No differences regarding the patients’ gender was observed in this study between the control group and the study group. However, patients with confirmed gastric adenocarcinoma were significantly older than the control group (54 vs. 67 yrs). No association of comorbidities with gastric cancer were observed even if there was an increasing trend in this group, as there were no significant differences. Our analysis showed that miR-106 expression levels are not influenced by age (rs = +0.215, *p* = 0.195) and also gender has no influence on them (rs = −0.078, *p* = 0.648). Median values of fold change in the confirmed adenocarcinoma group were significantly higher as the Mann–Whitney U test shows (Figure 1).

### 2.1. Higher Expressions Levels of miR-106 in Gastric Juice Are Corelated with CEA Levels

In our comprehensive investigation into tumoral markers, we delved deeper into the relationship between miR-106 levels and two commonly studied biomarkers, CA 19-9 and CEA. Our findings revealed a noteworthy correlation: between miR-106 expression and the CA 19-9 marker, there are no correlations (r_s_ = +0.241, *p* = 0.145). On the other hand, CEA (r_s_ = +0.406, *p* = 0.011) shows good correlation. However, our analysis went further by employing multivariable linear regression, where adjustments were made for age and gender variables. Surprisingly, CEA emerged as a robust predictor of elevated miR-106 expression levels (Table 2). This highlights the unique predictive capacity of CEA in influencing miR-106 expression levels.

### 2.2. Comparisons between miR-106 Levels, CEA, CA 19-9 in Predicting Tumor Extension, Nodal Involvement and Metastases

**Figure 2:**
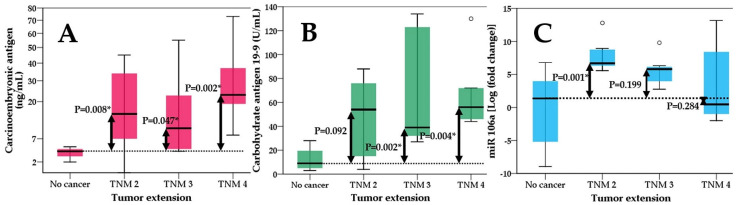
Kruskal–Wallis test showing differences between non-cancerous group and different stages of tumor extension (TNM 2-4) for (**A**)—carcinoembryonic antigen; (**B**)—carbohydrate antigen 19-9 and (**C**)—miR-106; significant values are flagged with *.

For the next analysis, we split the patients according to the TNM stage as follows: No cancer—Group 0; TNM stage 2—Group 1; TNM stage 3—Group 2; TNM stage 4—Group 3 (the study had no stage 1 TNM patient). We ran the Kruskal–Wallis test and compared the results for each tumoral marker (CEA, CA 19-9 and the novel marker miR-106). The results showed that overall, the Kruskal–Wallis test was significant for all three markers. However, for miR-106a, the levels vary only between stage 2 TNM and the no cancerous patients group (*p* = 0.001) while no differences were seen between other categories, as shown in Figure 2. Interestingly, the mean values for each stage decreased as the cancer advanced. On the other hand, CEA and CA 19-9 showed significant differences among all stages compared with the non-cancerous group for CEA and for stage 3 and 4 TNM for the CA 19-9 marker (Figure 2). We could not find any differences among stages of gastric adenocarcinoma for each marker, only when the stages were compared to the non-cancerous control.

**Figure 3:**
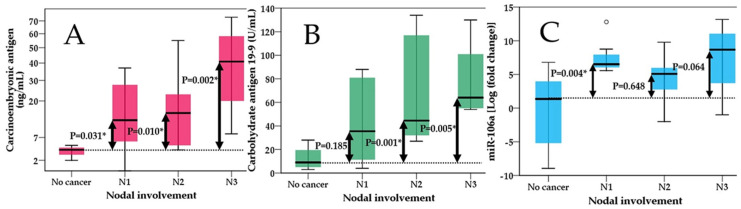
Kruskal–Wallis test showing differences between non-cancerous group and different stages of nodal involvement (N1-N3) for (**A**)—carcinoembryonic antigen; (**B**)—carbohydrate antigen 19-9 and (**C**)—miR-106; significant values are flagged with *.

Consecutively, we ran the same algorithm but this time we segregated patients based on nodal involvement as follows: Group 0—No cancer; Group 1—N1 stage; Group 2—N2 stage and Group 3—N4 stage. We executed the Kruskal–Wallis test again and the results showed overall significance for all three markers. Subgroup analysis revealed that for miR-106 levels, there were significant differences only between stage N1 and the non-cancerous group, while the rest were not significant (Figure 3). Markers like CEA showed significant differences between the non-cancerous group and all 3 stages of nodal involvement (N1-3). CA 19-9 also showed significant differences across all stages compared with the non-cancerous group except for the N1-stage. All three tests could not differentiate the degree of nodal involvement since all pairwise comparisons among cancerous groups were not significant (Figure 3).

**Figure 4:**
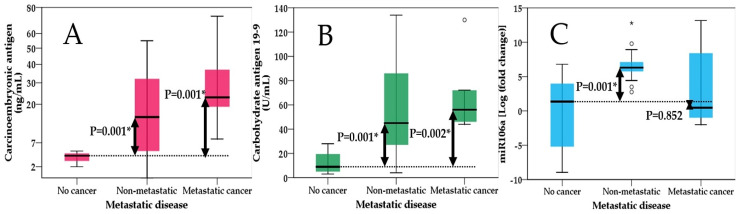
Kruskal–Wallis test showing differences between non-cancerous group, non-metastatic (TNM 2-3) and metastatic disease (TNM 4) for (**A**)—carcinoembryonic antigen; (**B**)—carbohydrate antigen 19-9 and (**C**)—miR-106; significant values are flagged with *. (Corrected to reflect accurate group assignments after data revision. One patient with TNM stage 2 was mistakenly included in the metastatic group, and one patient with TNM stage 4 was erroneously categorized as non-metastatic. The corrected group assignments align with revised dataset analyses).

Finally, we analyzed the presence of metastases by comparing the values of each markers. This time, patients were grouped as follows: Group 0—non-cancerous group; Group 1—non-metastatic tumor and Group 2—metastatic tumor. The Kruskal–Wallis test was significant for all three markers. Further analysis showed that each individual maker does not prove any significant differences among non-metastatic and metastatic disease, but can distinguish between non-cancerous and cancerous patients whether they are non-metastatic/metastatic, except for miR-106, which showed no significant differences in the mean ranks of metastatic and non-cancerous patients (Figure 4).

### 2.3. Comparative Analysis of miR-106, CEA, and CA 19-9 as Predictive Biomarkers for Positive Histopathological Examination in Gastric Cancer

**Figure 5:**
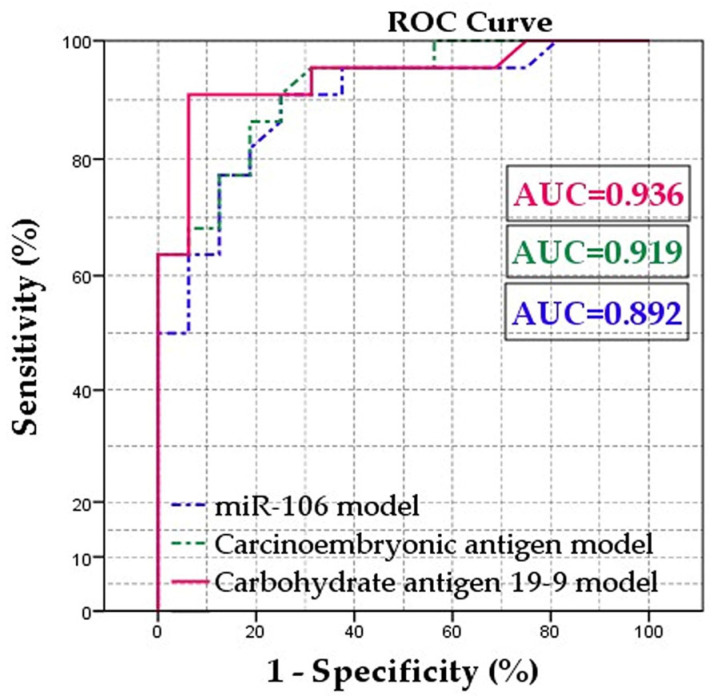
Receiver operating curve (ROC) showing the area under the curve (AUC) for the predicted probability given by the multivariable models containing the tumoral markers; highest AUC indicates really good accuracy of the multivariable model in predicting the positive histopathological exam. Adjustment for age and gender were made for each multivariable model.

To establish a hierarchy among miR-106, CEA and CA 19-9 as diagnostic markers for gastric adenocarcinoma, we conducted a comparative analysis using multivariable models adjusted for age and gender. Initially, we generated receiver operating characteristic (ROC) curves and calculated the area under the curve (AUC) for miR-106, CEA and CA 19-9: 0.818—*p* < 0.001; 0,903—*p* < 0.903 and 0.902—*p* < 0.001, respectively. The determined cut-off points for miR-106, CEA, and CA 19-9 were 4.3 (sensitivity 79.3%, specificity 81.3%), 4.5 ng/mL (sensitivity 86.4%, specificity 75%) and 26.5 U/mL (sensitivity 81.8%, specificity 93.7%), respectively (Figure 5).

Next, we categorized patients based on these cut-off points into high or low tumoral marker value groups and conducted logistic regression models. The univariable and multivariable logistic regression analysis revealed that higher values of miR-106, CEA and CA 19-9 were predictive of a positive histopathological exam [OR:12,032 (CI 95% 1948–74,305)]; [OR: 30 (CI 95% 3141–286,576)] and, respectively, [OR: 55,866 (4512–691,687)] (Table 3). Subsequently, we saved the predicted probability of each case from the regression models and constructed ROC curves to determine which model offered the highest probability of a positive adenocarcinoma diagnosis. Our findings indicated that when considering age and gender, CA 19-9 emerged as the best predictor of a positive histopathological exam for gastric adenocarcinoma, with an AUC of 0.936 (*p* < 0.001), (Table 4).

We determined that our study achieved a statistical power of 0.83. The significance level (alpha) was set at 0.05. These results indicate that our sample size of 38 patients provides adequate power to detect significant effects within the parameters of our study design. Specifically, our power analysis suggests that we have an 83% probability of detecting a true effect if one exists, at a significance level of 0.05.

## 3. Discussions

Our prospective study conducted at a single center included 38 patients, comprising 22 individuals with confirmed gastric adenocarcinoma and 16 non-cancerous cases. The aim was to investigate whether miR-106 could serve as a diagnostic marker. Despite notable progress in gastric cancer (GC) research, our understanding of the molecular mechanisms driving cancer invasion and metastasis remains limited. This deficiency underscores the urgency for further exploration. Early-stage GC often presents without noticeable symptoms, resulting in delayed diagnosis until the disease has advanced or metastasized distantly [31,32,33].

Our results show that miR-106 from the gastric juice is a good surrogate marker with an AUC over 0.8 in detecting gastric adenocarcinoma (Figure 1). Since our study calculated the log_2_ (fold change miR-106) it is necessary to elucidate the actual value of the increased fold as follows: 2^(4,3)^ = 19.7. Therefore, for a value of the fold change greater than 19.7 compared to the average control group, there is nearly 92.3% (66–98.6%) probability of positive diagnostic of gastric adenocarcinoma. Previous studies have demonstrated that miR-106 levels are elevated in gastric juice samples obtained from patients who have been diagnosed with gastric cancer. This finding suggests that miR-106 could serve as a promising biomarker for the detection and monitoring of gastric cancer [32]. In a systematic review by Ahadi, miR-106 from 574 tissue sample were analyzed and the mean fold change was 2.8 (1.52–9.02) [34]. These results are very different from ours, suggesting that the expression of miR-106 in the gastric juice could be higher. Despite numerous studies indicating significant associations between miR-106a expression levels and various clinicopathological features of gastric cancer, such as tumor stage, size, differentiation, lymphatic and distant metastasis and invasion [23,31,32,33,34], our subsequent analysis using the Kruskal–Wallis test revealed a significant overall difference (*p* < 0.001) across all clinical features, including nodal involvement, metastases and tumor expansion (TNM staging). However, upon pairwise comparison, no significant differences were observed except between cancerous and non-cancerous groups. MiR-106, known for its oncogenic properties, is frequently found at elevated levels in both tissue samples and peripheral blood from individuals with gastric cancer. This observation suggests that miR-106 could serve as a valuable biomarker for the detection and monitoring of circulating tumor cells (CTCs) in gastric cancer patients [35]. A study discovered that miR-106 is often upregulated in early-stage gastric cancer, where it promotes cell proliferation by targeting and downregulating the tumor suppressor gene p21 [36]. Liu et al. proposed the intriguing concept that gastric cancer cells exhibit a dual role in miRNA dynamics—they not only release certain cellular miRNAs into the extracellular environment but also selectively uptake specific miRNAs. This bidirectional process suggests a complex interplay between cancer cells and their microenvironment, potentially influencing the composition and function of circulating miRNAs in gastric cancer patients [37]. Our study observed a similar trend when comparing miR-106 levels among patients stratified by tumor extension (TNM stage). This observation suggests a potential association rather than definitive corroboration. Notable distinctions were detected between non-cancerous patients and those in TNM stage 2, indicating a potential role of miR-106 in early-stage disease. However, as the cancer progresses to stage 3 and stage 4, these differences diminish. We assume that the observed attenuation in miR-106 levels in advanced stages may be attributed to a feedback mechanism wherein cancer cells cease the release of certain miRNAs and instead actively uptake them for oncogenic purposes, as studies showed that miR-106 is an important element in tumor invasion and metastasis [32]. However, due to the small number of cases with advanced disease, the significance of reduced miR-106 expression in this context remains uncertain. While we can hypothesize mechanisms involved in miR-106 downregulation in advanced disease stages, such as feedback mechanisms or altered uptake by cancer cells for oncogenic purposes, these conclusions are based on preliminary data. Larger-scale studies are needed to provide more robust evidence and clarify the role of miR-106 in advanced stages of gastric cancer.

The limited sample size in our study restricts definitive conclusions about the impact of miR-106 expression levels in advanced disease. Future research efforts should aim to include larger cohorts to validate these initial findings and uncover the underlying mechanisms driving miR-106 dysregulation in metastatic and advanced gastric cancer.

CEA, or carcinoembryonic antigen, is typically produced during fetal development in gastrointestinal tissue, but its production ceases before birth. As a result, CEA is normally present at very low levels in the blood of healthy adults, typically around 2–4 ng/mL [38]. Serum CEA serves as a representative tumor marker and is known to be elevated in various solid tumors, including colorectal cancer, gastric cancer, pancreatic cancer, pulmonary cancer, breast cancer and head and neck cancer. However, it is important to note that elevated serum CEA levels can also occur in non-cancerous conditions, such as alcoholic hepatic cirrhosis, chronic bronchitis, pulmonary emphysema, ulcerative colitis, gastric ulcer and atrophic gastritis. Additionally, CEA levels may be elevated in certain healthy individuals, such as the elderly or smokers [39,40,41]. Our study showed positive correlation of carcinoembryonic antigen with tumor extension, lymph node involvement and metastasis (Figure 2, Figure 3 and Figure 4). Similar findings were also observed in a study by Park et al., where CEA was not only correlated with lymphatic and distant metastasis but also with tumor depth; however, this aspect was not assessed in our study [42]. Another study by Sisik et al. also showed that both markers CEA and CA 19-9 showed good correlation especially in advanced stages of colorectal cancer and gastric cancer [43]. Our study uncovered a significant correlation between miR-106 levels in gastric juice and serum CEA levels, highlighting an important association (B-coefficient: 0.175; CI 95%—0.025–0.326) (Table 2). However, we did not observe a similar correlation with CA 19-9 levels, age or gender suggesting distinct dynamics between miR-106 and CA 19-9, and we also did not observe any influences from demographics.

Carbohydrate antigen 19-9, also referred to as sialyl-Lewis A, is a tetrasaccharide typically found attached to O-glycans on cell surfaces. Its primary function involves facilitating cell-to-cell recognition processes [44]. Our findings indicate that CA 19-9 may serve as the most effective predictive marker for a positive histopathological exam of gastric adenocarcinoma. Notably, a separate study demonstrated that postoperative positive values of CA 19-9, but not CEA, could predict recurrence [45]. It is worth highlighting that while CA 19-9 may demonstrate superior predictive performance in specific scenarios, our analysis indicates that both CA 19-9 and CEA possess robust predictive capabilities. The area under the curve (AUC) values for each model revealed excellent predictive probabilities, especially when the marker values surpassed the designated cut-off points (Table 3). Furthermore, considering the odds ratios (ORs), we calculated the probabilities of confirmed cases for CEA levels exceeding 4.5 ng/mL and CA 19-9 levels surpassing 26.5 U/mL. These calculations yielded percentages of confirmed cases as follows: for CEA, 96.8% (with a confidence interval of 75.9–99.7%) and for CA 19-9, 98.2% (with a confidence interval of 81.9–99.8%). These findings underscore the strong predictive potential of both CEA and CA 19-9 in identifying cases of gastric adenocarcinoma.

Our novel approach aimed to assess whether these markers could discern significant differences among various histopathological features. Surprisingly, we found that neither CEA nor CA 19-9 could distinguish between different stages of cancer, only showing significant differences when compared to the non-cancerous group. Notably, miR-106 levels in gastric juice from advanced cases (TNM 3 and 4) were not significantly different from those of non-cancerous patients, suggesting that miR-106 may exhibit more modest performance compared to the other markers previously examined. This observation was also exhibited in one meta-analysis on miR-106 and its ability to detect cancer, which showed overall modest performance for both colorectal and gastric cancer [46].

While our results show that CEA predicts the levels of miR-106 in linear regression, further studies are needed to assess if combining protein biomarkers with miRNAs is a novel and promising strategy for cancer detection and prediction. For instance, miR-141 and CEA are well-established biomarkers in colorectal cancer (CRC), where studies have demonstrated that their combined use enhances sensitivity compared to individual markers alone, thereby reducing the misdiagnosis rate among CRC patients. The applicability of these findings to gastric adenocarcinoma (GA) requires further exploration [47].

## 4. Materials and Methods

The research was conducted in Sibiu Clinical Emergency Hospital (Gastroenterology Department) in association with the Molecular Biology Laboratory of the Applied Ecology Research Center from Lucian Blaga University of Sibiu, Romania. To attain the established objectives, a clinical study was conducted, focusing on the quantitative analysis of microRNA results (from gastric juice) detected by RT-qPCR, correlated with the endoscopic diagnosis of gastric lesions and the histopathological result of biopsies in the tumoral stage (depth of the malign lesion was established with endoscopic ultrasound).

Inclusion criteria established were:

1. Adult participants, aged 18 years and older;
2. Patients diagnosed with adenocarcinoma at various stages, with histopathological biopsy results confirming the diagnosis;
3. Patients with an endoscopic description of the lesion.

Biopsies from patients diagnosed with minimal superficial gastritis (n = 10) and healthy individuals (n = 6) were utilized as controls in the study, providing histopathological results for comparison.

For tumor staging, we used endoscopic ultrasound based on AJCC (American Joint Committee on Cancer) T staging 8th edition (described the tumor invasion in depth, considering the involved layer of the gastric wall): T1 (tumor invades submucosa), T2 (tumor invades muscularis propria), T3 (tumor penetrates subserosa connective tissue without invasion of visceral peritoneum), T4 (invasion in other organs or large vessels).

Exclusion criteria consisted of:

1. Patients with other associated malignancies;
2. Prior chemotherapy for gastric cancer.

Gastric juice was aspired during endoscopy before taking tissue biopsies. In this study, we used gastric juice (endoscopically aspirated) from 38 patients: 22—the study group (with gastric adenocarcinoma) and 16—the non-cancerous control group. Liquid biopsy simplifies the drawbacks of traditional biopsy techniques and addresses issues related to tumor tissue heterogeneity by facilitating the analysis of genetic markers in bodily fluids.

Demographic attributes, encompassing age and gender, were meticulously collected alongside pertinent clinical information, including concurrent health conditions such as diabetes and cardiovascular diseases (hypertension and ischemic heart disease). Additionally, clinicopathological features pertaining to the tumor, such as TNM stage, lymph node involvement and metastatic disease, were rigorously extracted from medical records and comprehensively reviewed. The laboratory assessment involved the analysis of miR-106, CEA, CA 19-9, hematological markers and biochemical parameters such as CRP and LDH. All data were gathered from electronic medical records and meticulously reviewed by seasoned clinicians.

### 4.1. MiRNA Extraction and Quantification

Total RNA was extracted from gastric juice using the TRIzol Reagent (Invitrogen/Thermo Fisher Scientific, Waltham, MA, USA) following the manufacturer’s recommendation. For every patient, miRNA was extracted from 500 μL of gastric juice and eluted in a final volume of 40 μL of ultrapure water (Invitrogen). MiR-106’s quantity and quality of purified RNA was assessed by Qubit with the Qubit microRNA assay kit (Invitrogen by Thermo Fisher Scientific). MiR-106 (Hsa-miR-106a-5p) levels in gastric juice were quantified from extracted RNA by reverse-transcription followed by quantitative PCR. An equal mass of miRNA was reverse transcribed using the TaqMan microRNA reverse transcription kit (Invitrogen/Thermo Fisher Scientific) following the manufacturer’s protocol. Briefly, 5 μL RNA was mixed with 7 μL RT mix and 3 μL of primer specific for miR-106 and incubated at 16 °C for 30 min followed by 42 °C for another 30 min. The reaction was inactivated by 5 min incubation at 85 °C. For specific reverse transcription and amplification of miR-106, we used the recommended TaqMan assay (Thermo Fisher Scientific). Following reverse transcription, miR-106 levels were quantified by real-time PCR using the Taqman universal master mix II, no UNG, and the specific Taqman microRNA assay (Thermo Fisher Scientific). The mix was incubated at 95 °C for 10 min for enzyme activation followed by 40 cycles of amplification (95 °C for 15 s and 60 °C for 1 min). U6 RNA was quantified in parallel with the miR-106NA using a specific TaqMan assay (Thermo Fisher Scientific) for this RNA and was used for normalization. To establish U6 as a reliable standard for normalization, we conducted both a *t*-test and Levene’s test for homogeneity of variance to ensure equality of U6 expression across groups. Our preliminary data analysis revealed consistent results: the t-test yielded a non-significant difference in means (t(36) = 1.080, *p* = 0.288), indicating similar U6 expression levels between groups. Additionally, Levene’s test showed no significant differences in variances (F(36) = 0.134, *p* = 0.716). These findings confirm that U6 expression remains consistent across both groups, validating its suitability as a normalization standard. The procedure involved in calculating the fold change value of miR-106 through normalization to U6 followed these steps.

First, the ΔCt value was determined using the formula ΔCt = Ct(miR-106) − Ct(U6), where Ct represents the cycle threshold obtained from qPCR analysis. Subsequently, ΔΔCt was calculated to ascertain the relative expression level. This involved subtracting the average ΔCt value of the control group from the ΔCt value of each sample, denoted as ΔΔCt = ΔCt − average ΔCt in controls. The relative fold change value was then derived using the formula fold change = 2^−ΔΔCt^. Due to the skewed distribution of fold change values, a log-transformation was applied to express the levels of fold change for miR-106. This was achieved by taking the base 2 logarithm of the fold change values, expressed as log2 (miR-106-fold change). To determine the optimal cut-off point, the log-transformed fold change values were reverted to their original scale by reversing the log transformation. We chose the relative expression of miR-106 normalized to U6 by comparing the study group values with the average control group values because the Ct values of U6 were overall lower compared to the miR-106 levels (meaning higher expression of U6), therefore analyzing and interpreting data normalized to a higher expression normalizer requires careful consideration and may involve more complex statistical methods to accurately assess differences between groups while accounting for normalization biases.

For each qPCR assay performed in our research, we included triplicate technical replicates. This approach ensured robustness and reliability in our quantitative measurements of gene expression levels. The mean of these triplicates was used for statistical analyses and interpretation of results.

### 4.2. Statistical Analysis

This study utilized various statistical methods to analyze different types of data. Continuous variables were described using the median and interquartile range and differences between groups were assessed using the Mann–Whitney U test. Categorical variables were presented as counts and percentages and Fisher’s exact test was employed to examine differences between groups. Normality of the data was assessed using the Shapiro–Wilk test and, due to skewed distributions, correlation analysis was conducted using the Spearman coefficient. Receiver operating characteristic (ROC) analysis was utilized to calculate the area under the curve (AUC) for predictive markers, with the optimal cut-off points determined using the Youden’s J index, which combines sensitivity and specificity. Linear regression was employed to identify variables predicting the levels of specific markers, with B-coefficients reported. Differences in mean ranks of different markers for each clinical feature were assessed using the Kruskal–Wallis test. Univariable and multivariable regression analyses were conducted to identify predictive variables for a positive histopathological exam, with odds ratios (ORs) presented. For each multivariable model, a new variable representing the predicted probability was created and the goodness-of-fit of each model was assessed by calculating the AUC through ROC analysis. The model with the highest AUC was considered the most accurate for prediction. Confidence intervals of 95% were reported for odds ratios, AUC and B-coefficients. The significance level (alpha) was set at 0.05. Statistical analysis was performed using IBM-SPSS version 22.0.

## 5. Conclusions

In conclusion, our study underscores the robust predictive capabilities of CEA and CA 19-9 in diagnosing gastric adenocarcinoma, outperforming miR-106, which exhibits more modest performance. This observation, coupled with the reported dynamics of miR-106 in gastric juice from previous studies, highlights the need for further research to elucidate its role and potential clinical applications in gastric cancer diagnosis. These findings emphasize the importance of comprehensive biomarker approaches in improving the diagnostic accuracy and clinical management strategies for gastric adenocarcinoma.

## 6. Limitation of the Study

This study may be limited by several factors. Firstly, the sample size was relatively small, which could impact the generalizability of the findings. Conducting the study at a single center may have restricted the diversity of patient populations and clinical settings included in the analysis. Additionally, the cross-sectional design of the study may hinder the establishment of causal relationships between miR-106, CEA, CA 19-9 levels and gastric adenocarcinoma.

## Figures and Tables

**Table 1:** General characteristics of the patients.

Variable		Normal Values	Control Group	Adenocarcinoma Group	*p*-Value
**Demographic characteristics**					
Gender	Male		7 (31.8%)	15 (68.2%)	0.132
	Female		9 (56.2%)	7 (43.8%)	
Age			54 (±12.8)	67 (±11.2)	0.002
**Significant comorbidities**					
Diabetes			2 (12.5%)	5 (22.7%)	0.645
Cardiovascular disease			3 (18.7%)	10 (45.4%)	0.086
No comorbidities			0	2 (9.1%)	0.215
**Laboratory assessment**					
Hemoglobin levels g/dL		12–15	13.5 (±0.8)	8.1 (±1.9)	<0.001
Hematocrit %		40–47	44 (±1.5)	33(±5)	<0.001
MCHC, g/dL		32–36	34 (±0.9)	28 (±4)	<0.001
MCV, fL		80–100	88 (±6)	73 (±6)	<0.001
CRP, mg/L		<5	2.6 (±1.1)	24.6 (±14.3)	<0.001
LDH, U/L		130–230	153 (±23)	265 (±64)	<0.001
ALP, U/L		20–140	130 (±18)	142 (±49)	0.324
CEA, ng/mL		<3	3.8 (±0.9)	21 (±19)	<0.001
miR-106a (fold change)			2.7 (0.04–15.9)	71.3 (21.5–343.7)	<0.001
CA 19-9 U/mL		0–27	11.7 (±8.7)	57.9 (±40)	<0.001

Significant values are flagged with green; ALP (alkaline phosphatase); CEA (carcinoembryonic antigen); CA 19-9 (carbohydrate antigen 19-9); CRP (C reactive protein); LDH (lactate dehydrogenase); MCHC (mean corpuscular hemoglobin concentration); MCV (mean corpuscular volume).

**Table 2:** Multivariable analysis of factors influencing miR-106 levels.

			95.0% Confidence Interval for B	
Variable	B	*p*-Value	Lower Bound	Upper Bound
Gender	2.274	0.236	−1.561	6.11
Age	0.008	0.918	−0.147	0.163
Carcinoembryonic antigen (CEA)	0.175	0.024	0.025	0.326
Carbohydrate antigen 19-9 (CA 19-9)	−0.01	0.779	−0.078	0.059

Significant values are flagged with green.

**Table 3:** Univariable/multivariable regression of the markers predicting a positive histopathological exam.

	Univariable Regression				Multivariable Regression			
	*p*-Value	Odds Ratio (OR)	95% CI for OR		*p*-Value	Odds Ratio (OR)	95% CI for OR	
Variable			Lower	Upper			Lower	Upper
miR-106	0.001	14,733	2,965	73,208	0.007	12,032	1948	74,305
CEA	0.001	19	3604	100,154	0.003	30	3141	286,574
CA 19-9	<0.001	67.5	6795	670,529	0.002	55,866	4512	691,687

miR-106—micro-RNA 106; CEA—carcinoembryonic antigen; CA 19-9—carbohydrate antigen 19-9.

**Table 4:** Area under the curve of the predicted probabilities given by the multivariable models.

Variable	Area	Std. Error	*p*-Value	Confidence Interval 95%	
				Lower Bound	Upper Bound
miR-106 model	0.892	0.052	<0.001	0.789	0.995
CEA model	0.919	0.043	<0.001	0.836	1.000
CA 19-9 model	0.936	0.041	<0.001	0.856	1.000

miR-106—micro-RNA 106; CEA—carcinoembryonic antigen; CA 19-9—carbohydrate antigen 19-9.

## Data Availability

The datasets generated and analyzed during the current study are not publicly available due to institutional restrictions, but are available from the corresponding author upon reasonable request.
